# OQ-FERNet: An occlusion-aware quantitative facial expression recognition network for real-time human-computer interaction

**DOI:** 10.1371/journal.pone.0359554

**Published:** 2026-10-01

**Authors:** Lianfei Gao, Junfeng Tan, Jiahao Zhang

**Affiliations:** Shandong Huitong Financial Leasing Co., Ltd., Jinan, Shandong, China; National Changhua University of Education, TAIWAN

## Abstract

Facial expression recognition is an important task in real-time human-computer interaction, but its practical application is still limited by partial facial occlusion, high computational cost, and the lack of fine-grained emotion representation. To address these issues, this paper proposes OQ-FERNet, an occlusion-aware quantitative facial expression recognition network. The proposed network adopts MobileNetV4-Conv-S as a lightweight facial expression feature extraction backbone to obtain compact and discriminative representations with low computational cost. On this basis, an Occlusion-aware Regional Reweighting module is designed to divide the feature map into upper-face, middle-face, and lower-face regions, and adaptively adjust their contributions according to regional reliability. This design suppresses occluded or less informative facial areas while enhancing visible expression-related cues. Furthermore, a Quantitative Emotion Prediction Head is introduced to jointly perform discrete expression classification and emotion intensity estimation, enabling the model to provide both categorical and fine-grained affective outputs. Experiments are conducted on RAF-DB and AffectNet-7. The results show that OQ-FERNet achieves competitive classification performance, improved robustness under different occlusion conditions, and effective quantitative emotion prediction. Specifically, OQ-FERNet obtains 93.12% accuracy on RAF-DB and 68.32% accuracy on AffectNet-7, while achieving an MAE of 0.246, an MSE of 0.101, and an RMSE of 0.318 for quantitative emotion prediction on AffectNet-7. In addition, the model contains only 4.18M parameters and 0.26G FLOPs, with an inference speed of 356 FPS. These results indicate that OQ-FERNet provides an effective and efficient solution for lightweight, occlusion-robust, and fine-grained facial expression recognition in real-time human-computer interaction scenarios. The source code is publicly available at: https://github.com/jiahao001-j/OQ-FERNet.

## Introduction

Facial expression recognition (FER) is an important research topic in affective computing and computer vision, aiming to enable machines to interpret human emotional states from facial appearance. As a natural and intuitive form of non-verbal communication, facial expressions provide essential cues for understanding human intention, affective response, and interaction behavior. In recent years, FER has attracted increasing attention in human-computer interaction, intelligent education, driver monitoring, healthcare assistance, and service robotics [1–3]. With the development of deep learning, especially convolutional neural networks (CNNs), FER methods have gradually shifted from handcrafted feature extraction to end-to-end representation learning. Compared with traditional descriptors such as local binary patterns, histogram of oriented gradients, and scale-invariant features, deep neural networks can automatically learn hierarchical facial features and have achieved promising performance on several benchmark datasets [4].

Existing FER studies have mainly focused on improving the discriminative ability of facial feature representations. Early deep learning-based methods commonly adopted classic CNN architectures, such as VGG, ResNet, and Inception, to extract global facial features for expression classification [5]. Subsequently, attention mechanisms, multi-scale feature fusion, region-based learning, and spatial-temporal modeling were introduced to capture subtle facial muscle variations and improve recognition accuracy in unconstrained environments [6–8]. These studies have significantly promoted the development of FER and demonstrated the strong capability of deep models in representing complex facial patterns. However, as FER research moves toward real-time human-computer interaction scenarios, model efficiency becomes increasingly important. Many high-performance networks rely on deep backbones or additional complex modules, which may lead to large parameter sizes, high computational costs, and increased inference latency [9,10]. Therefore, lightweight FER models that can maintain competitive recognition performance while reducing computational complexity are still needed for real-time deployment.

Lightweight neural networks provide a practical direction for efficient visual recognition. Representative architectures such as MobileNet, ShuffleNet, and EfficientNet-lite reduce computational cost through depthwise separable convolution, inverted residual structures, channel shuffle operations, or compound scaling strategies [11,12]. These designs have been widely used in mobile and edge vision tasks. Recently, MobileNetV4 has further improved the efficiency-performance trade-off through more effective lightweight convolutional designs, making it suitable for real-time visual perception tasks [13]. For FER, lightweight backbones can reduce inference cost and improve deployment flexibility. Nevertheless, directly applying a lightweight classification network to FER may not fully consider the special characteristics of facial expressions [14]. Facial expression cues are usually distributed in local regions such as the eyebrows, eyes, cheeks, mouth corners, and lips. Excessive downsampling or insufficient regional modeling may weaken fine-grained expression details, especially in compact networks. This motivates the design of lightweight FER models that preserve efficient feature extraction while enhancing the use of expression-related facial regions. Another important research direction in FER is robustness under unconstrained conditions. In practical scenarios, facial images may be affected by head pose changes, illumination variation, motion blur, low resolution, and partial occlusion [15]. Among these factors, occlusion is particularly common because facial regions may be covered by masks, glasses, hands, hair, or other objects [16,17]. Region-based FER studies have shown that different facial areas contribute differently to expression perception. For example, the upper face is closely related to eyebrow and eye movements, whereas the lower face contains important information from the mouth, lips, and chin. When one region is occluded or degraded, the model should rely more on visible and reliable regions rather than treating all spatial features equally. Existing attention-based and occlusion-robust methods have attempted to address this issue through spatial attention, part-based learning, or occlusion augmentation [18–20]. However, some methods introduce additional computational burden or require more complex training strategies. For real-time FER, a simple and efficient regional reweighting mechanism is more suitable for balancing robustness and efficiency.

In addition to classification accuracy, the form of emotional output is also an important aspect of FER. Most existing FER models follow a categorical emotion recognition paradigm, where each facial image is assigned to one of several basic emotion classes, such as happiness, sadness, anger, fear, surprise, disgust, or neutral. This paradigm is simple and widely used, but facial expressions in real interactions are often gradual rather than strictly discrete. For instance, two samples classified as happiness may correspond to different expression intensities, such as a slight smile and a strong joyful expression. Continuous emotion representations, such as valence-arousal models, provide a more detailed description of affective states, but they usually require more specific annotations and may increase the difficulty of model training [21,22]. Therefore, combining expression classification with a lightweight emotion intensity estimation branch can provide more informative outputs while maintaining the practicality of categorical FER.

Based on the above considerations, this paper proposes an Occlusion-aware Quantitative Facial Expression Recognition Network, named OQ-FERNet, for real-time human-computer interaction scenarios. The proposed method uses MobileNetV4-Conv-S as a lightweight backbone to extract facial expression features efficiently. To improve robustness against partial occlusion and local information degradation, a regional reweighting module is introduced to adaptively adjust the contributions of upper-face, middle-face, and lower-face regions. Furthermore, a multi-task prediction head is designed to output both expression categories and emotion intensity scores. In this way, the model aims to achieve efficient, robust, and more fine-grained facial expression recognition without introducing a complex interaction system or heavy temporal architecture. The present study focuses on facial expression recognition from static images. The lightweight frame-difference pathway shown in the overall architecture is retained as an auxiliary architectural extension for sequential video input, allowing adjacent-frame variation cues to be incorporated when temporally continuous frames are available. Since RAF-DB and AffectNet-7 consist of isolated facial images, the empirical evaluation reported in this paper concerns the static-image pathway.

The main contributions of this paper are summarized as follows:

- A lightweight FER model named OQ-FERNet is proposed for real-time human-computer interaction scenarios, using MobileNetV4-Conv-S as an efficient feature extraction backbone.
- An occlusion-aware regional reweighting module is designed to adaptively adjust the importance of upper-face, middle-face, and lower-face features, improving the model’s robustness to partial occlusion and local expression loss.
- A quantitative emotion prediction head is introduced to jointly perform facial expression classification and emotion intensity regression, enabling the model to provide more fine-grained emotional outputs.

The remainder of this paper is organized as follows. Section 2 reviews related work on deep learning-based FER, lightweight neural networks, and occlusion-robust multi-task FER methods. Section 3 introduces the proposed OQ-FERNet in detail. Section 4 presents the experimental settings, comparison results, ablation studies, and robustness analysis. Section 5 concludes this paper and discusses future work.

## Related work

### Deep learning-based facial expression recognition

Facial expression recognition (FER) has been extensively studied in affective computing and computer vision. Early FER methods mainly depended on handcrafted facial descriptors, such as local binary patterns, histogram of oriented gradients, Gabor features, and scale-invariant features, followed by traditional classifiers including support vector machines, random forests, or shallow neural networks [23,24]. These methods are relatively simple and interpretable, but their performance is often limited by the representation ability of manually designed features, especially under complex conditions such as illumination changes, pose variations, and expression ambiguity.

With the development of deep learning, convolutional neural networks (CNNs) have become the mainstream framework for FER. Compared with handcrafted methods, CNN-based models can automatically learn hierarchical facial representations from input images and have achieved improved performance on benchmark datasets such as CK + , FER2013, RAF-DB, AffectNet, and Oulu-CASIA [25,26]. Representative architectures, including VGG, ResNet, Inception, DenseNet, and Xception, have been widely adopted as feature extractors for facial expression classification. In addition, attention mechanisms and multi-scale feature fusion strategies have been introduced to enhance expression-related regions and capture subtle facial muscle variations [27].

Although deep learning-based FER methods have significantly improved recognition accuracy, many existing models still rely on relatively deep backbones or additional complex modules. This may increase computational cost and limit their deployment in real-time human-computer interaction scenarios. Therefore, efficient FER models that can balance recognition accuracy, robustness, and inference speed remain worthy of further investigation.

### Lightweight neural networks for real-time recognition

Lightweight neural networks have been extensively investigated for real-time and resource-constrained visual recognition tasks. Unlike conventional deep CNNs that improve accuracy mainly by increasing depth, width, or structural complexity, lightweight networks aim to reduce parameters and floating-point operations while preserving sufficient representation capability. MobileNet is one of the most representative lightweight architectures. It introduces depthwise separable convolution to decompose standard convolution into depthwise and pointwise operations, significantly reducing computational cost [28,29]. MobileNetV2 further introduces inverted residual blocks and linear bottlenecks, improving the efficiency and feature representation of compact models. MobileNetV3 combines neural architecture search and lightweight attention mechanisms to improve the accuracy-efficiency trade-off [30].

Besides MobileNet-based models, other lightweight architectures have also been proposed. ShuffleNet uses pointwise group convolution and channel shuffle operations to improve information exchange across channels while reducing computation [31]. EfficientNet adopts compound scaling to balance network depth, width, and input resolution, and its lightweight variants have been applied to mobile visual recognition tasks [32]. More recently, MobileNetV4 has further improved mobile vision models through efficient convolutional designs and universal inverted bottleneck structures, providing a stronger balance between accuracy and runtime efficiency on mobile and edge devices [33,34]. These lightweight networks provide an effective foundation for real-time FER. In the context of FER, lightweight networks are particularly valuable because facial expression recognition often needs to process continuous image or video frames. In applications such as online interaction, driver monitoring, and embedded affective perception, the model must respond quickly while using limited computational resources. Therefore, several studies have explored lightweight FER models based on MobileNet, ShuffleNet, EfficientNet-lite, and depthwise separable convolution [35,36]. These methods demonstrate that compact backbones can reduce inference cost and improve deployment feasibility.

Nevertheless, directly applying general lightweight classification networks to FER may overlook the region-specific nature of facial expressions. Discriminative expression cues are often concentrated in local areas such as the eyes, eyebrows, cheeks, and mouth. In compact networks, aggressive downsampling may weaken these subtle details, while generic backbones usually lack explicit modeling of regional importance. Therefore, lightweight FER models should not only reduce computational complexity but also enhance task-specific local expression representation. This motivates the use of MobileNetV4-Conv-S as an efficient backbone, together with a simple regional reweighting strategy for facial expression features.

### Occlusion-robust and multi-task facial expression recognition

Robustness is a key issue in FER, especially under unconstrained real-world conditions. Facial images collected in practical scenarios are often affected by head pose changes, illumination variation, motion blur, low image quality, and partial occlusion. Among these factors, occlusion is particularly important because it may directly cover expression-related facial regions. For example, masks may hide the mouth and chin, glasses may affect the eye region, and hands or hair may block local facial structures. Since different expressions depend on different facial areas, occlusion can cause incomplete or misleading expression information.

To improve occlusion robustness, researchers have explored several strategies. Data augmentation methods simulate occlusion during training by randomly masking facial regions, encouraging the model to learn more robust representations [37]. Part-based methods divide the face into local regions or patches and extract regional features separately, so that the model can still use visible regions when some areas are corrupted. Attention-based methods attempt to assign higher weights to informative facial regions and reduce the influence of occluded or irrelevant areas [38]. Region attention networks further show that adaptively modeling the importance of facial regions can improve FER performance under pose variation and occlusion [39]. These studies demonstrate the importance of regional modeling for robust FER. However, some occlusion-robust methods introduce additional branches, landmark guidance, complex attention maps, or heavy feature fusion modules [40]. Although these designs can improve robustness, they may also increase model complexity and reduce inference efficiency. For real-time FER, it is more practical to design a lightweight mechanism that can estimate regional importance without relying on pixel-level occlusion labels or complex restoration procedures. Efficient attention mechanisms, such as ECA-style one-dimensional convolution, provide a useful idea for generating adaptive weights with low computational overhead [41]. Based on this idea, regional reweighting can be used to enhance visible and reliable facial regions while suppressing low-quality regions.

In addition to robustness, multi-task learning has also been widely studied in affective computing. Facial expression analysis is closely related to action unit detection, valence-arousal estimation, engagement recognition, and emotion intensity prediction. By learning related tasks jointly, multi-task models can obtain more informative feature representations and improve generalization [42,43]. Most existing FER methods, however, still focus on categorical emotion classification. They assign each input face to a discrete emotion class, such as happiness, sadness, anger, fear, surprise, disgust, or neutral [44,45]. This categorical setting is simple and effective, but it cannot fully describe the gradual nature of emotional expression. In real interactions, the same emotion category may appear with different activation degrees. For example, a slight smile and a strong joyful expression may both be classified as happiness, but their emotional intensities are clearly different [46].

Continuous affective models, such as valence-arousal representations, provide a more fine-grained description of emotional states. Nevertheless, they usually require additional annotations and may increase training difficulty. For practical FER, a feasible solution is to combine expression classification with an auxiliary intensity regression task. However, jointly modeling categorical expressions and continuous affective states introduces a distinct design conflict in lightweight FER. Conventional multi-task architectures often employ high-dimensional shared representations, task-specific feature-fusion layers, or separate multi-layer prediction branches, which increase the number of parameters, FLOPs, memory access, and inference latency. In contrast, aggressively compressed lightweight backbones may discard subtle facial variations required for stable continuous regression, making it difficult to preserve both fine-grained affective representation and real-time efficiency on edge hardware. To address this trade-off, OQ-FERNet reuses a single compact representation produced by the lightweight backbone and regional reweighting module, and applies only shallow task-specific output layers for expression classification and emotion intensity regression. This shared-feature design avoids duplicating dense feature extraction branches while retaining complementary categorical and quantitative supervision.

## Method

### Overall architecture of OQ-FERNet

The overall architecture of the proposed Occlusion-aware Quantitative Facial Expression Recognition Network (OQ-FERNet) is illustrated in Fig 1. The model is designed for lightweight and robust facial expression recognition in real-time human-computer interaction scenarios. As shown in the figure, OQ-FERNet consists of three main components: a MobileNetV4-Conv-S backbone, an occlusion-aware regional reweighting module, and a quantitative emotion prediction head. Given an aligned facial image, the backbone first extracts compact facial expression features with low computational cost. When sequential video frames are available, the architecture additionally supports an auxiliary frame-difference pathway for incorporating adjacent-frame variation cues. Then, the occlusion-aware regional reweighting module divides the feature map into upper-face, middle-face, and lower-face regions, and adaptively adjusts their contributions to enhance reliable expression cues. Finally, the quantitative emotion prediction head uses the reweighted features to predict both the facial expression category and the corresponding emotion intensity score. For static-image input, the reweighted representation is directly used for prediction, whereas for sequential video input, the lightweight frame-difference feature can be conditionally introduced to supplement the current-frame representation without requiring a recurrent network or a computationally intensive temporal architecture. Since the empirical evaluations in this study are conducted on the isolated facial images of RAF-DB and AffectNet-7, the reported results correspond to the static-image pathway, while the frame-difference pathway constitutes a completed auxiliary architectural extension for incorporating short-term variation cues when sequential video frames are available. Through this design, OQ-FERNet aims to achieve an effective balance among recognition accuracy, occlusion robustness, and inference efficiency.

**Fig 1:**
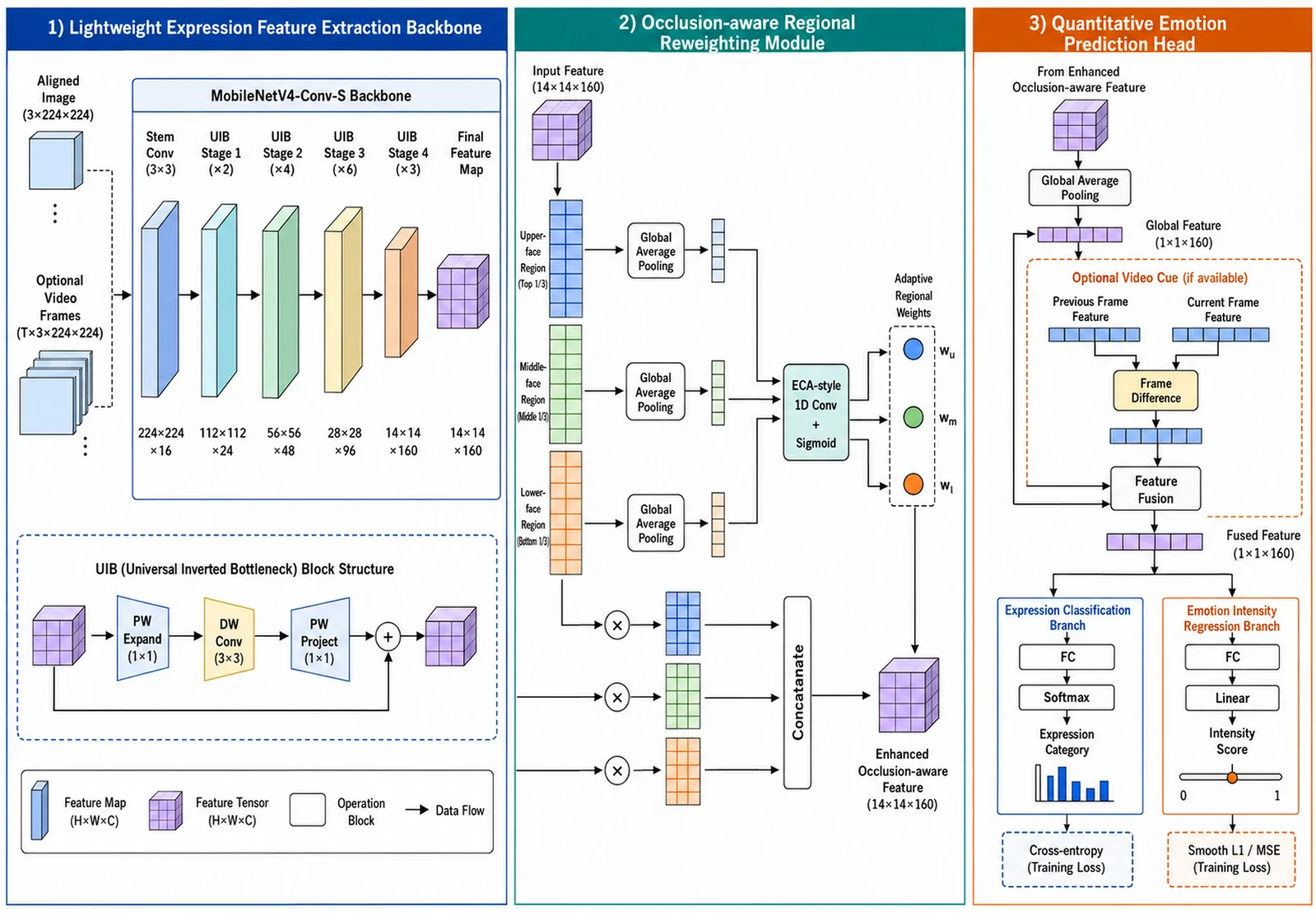
OQ-FERNet model overall architecture diagram.

### Lightweight expression feature extraction backbone

To meet the requirements of real-time facial expression recognition in human-computer interaction scenarios, MobileNetV4-Conv-S is adopted as the lightweight feature extraction backbone of OQ-FERNet. Compared with conventional deep convolutional networks, MobileNetV4-Conv-S has the advantages of low parameter complexity, reduced computational cost, and efficient feature representation. These properties make it suitable for real-time or edge-device facial expression recognition tasks.

Given an aligned facial image *X*, the backbone first extracts shallow facial texture features through the initial convolutional layers:


$$\begin{array}{c} F_{0}={Conv}_{s}(X) \end{array}$$ (1)

where *F*_0_ denotes the shallow expression feature, and ${Conv}_{s}(\cdot )$ represents the initial shallow convolution operation. Since facial expressions are usually caused by local muscle movements, shallow features contain important edge, texture, and local deformation information. Therefore, preserving fine-grained facial details in early stages is important for recognizing subtle expression cues around the eyes, eyebrows, mouth corners, and lips.

In the deeper stages, MobileNetV4-Conv-S mainly relies on the Universal Inverted Bottleneck (UIB) structure for efficient feature transformation. Let $F_{l-1}$ be the input of the *l*-th feature extraction stage. The output of the UIB module can be formu*l*ated as:


$$\begin{array}{c} F_{l}={UIB}_{l}(F_{l-1})={PW}_{project}\left({DW}_{k\times k}\left({PW}_{expand}(F_{l-1})\right)\right)+\delta (F_{l-1}) \end{array}$$ (2)

where ${PW}_{expand}(\cdot )$ denotes the point-wise convolution for channel expansion, ${DW}_{k\times k}(\cdot )$ denotes the depth-wise convolution with kernel size k×k, and ${PW}_{project}(\cdot )$ denotes the point-wise convolution for channel projection and fusion. The term δ(·) represents the residual connection, which is used only when the input and output feature dimensions are consistent. Through this inverted bottleneck design, the network first enhances feature representation in a higher-dimensional channel space, then captures local spatial patterns using depth-wise convolution, and finally compresses the channels to reduce computational cost.

Depth-wise separable convolution is an important factor that enables the lightweight property of MobileNetV4-Conv-S. For a feature map with spatial size H×W, input channels $C_{in}$, output channels $C_{out}$, and convolution kernel size k×k, the computational cost of standard convolution can be expressed as:


$$\begin{array}{c} C_{standard}=H\times W\times C_{in}\times C_{out}\times k\times k \end{array}$$ (3)

In contrast, the computational cost of depth-wise separable convolution is:


$$\begin{array}{c} C_{ds}=H\times W\times C_{in}\times k\times k+H\times W\times C_{in}\times C_{out} \end{array}$$ (4)

It can be observed that depth-wise separable convolution decomposes standard convolution into spatial filtering and channel fusion, thereby greatly reducing the computational burden of the backbone. This design allows MobileNetV4-Conv-S to extract effective facial expression features with lower complexity, which is consistent with the efficiency requirement of real-time FER.

After several UIB-based feature extraction stages, the backbone can generate multi-stage facial expression representations:


$$\begin{array}{c} \{F_{1},F_{2},...,F_{L}\}=Backbone(X),\;F_{b}=F_{L} \end{array}$$ (5)

where shallow features contain more local texture and fine-grained deformation information, while high-level features provide stronger semantic representation. In this study, the final high-level feature $F_{b}$ is used as the basic expression feature for subsequent processing. Although the lightweight backbone provides efficient feature extraction, different facial regions may contribute unequally under occlusion, pose variation, or local expression degradation. Therefore, regional modeling is further performed on $F_{b}$ to enhance reliable facial cues and reduce the interference of occluded or low-quality regions.

### Occlusion-aware regional reweighting module

In practical facial expression recognition scenarios, facial images are often affected by masks, glasses, hands, hair, head pose variation, or other forms of partial occlusion. These factors may cause incomplete or unreliable local expression information. Since different facial regions contribute differently to expression recognition, directly using the global feature extracted by the backbone may introduce interference from occluded or low-quality regions. To address this issue, an occlusion-aware regional reweighting module is designed to adaptively model the contributions of the upper-face, middle-face, and lower-face regions.

Let $F_{b}\in {\mathbb{R}}^{C\times H\times W}$ denote the basic facial expression feature extracted by the lightweight backbone, where *C*, *H*, and *W* represent the channel number, height, and width of the feature map, respectively. Since the input face image has been detected and aligned, the spatial structure of the feature map still maintains a certain correspondence with facial regions. Therefore, $F_{b}$ is divided along the height dimension into three regional features:


$$\begin{array}{c} \left\{F_{u},F_{m},F_{d}\}=Split(F_{b}\right) \end{array}$$ (6)

where $F_{u}$, $F_{m}$, and $F_{d}$ represent the upper-face, middle-face, and lower-face features, respectively. The upper-face region mainly contains expression cues from the eyebrows, eyes, and eye corners; the middle-face region reflects local variations around the nose, cheeks, and nasolabial area; and the lower-face region contains important information from the mouth corners, lips, and chin. This regional division enables the model to describe different facial areas separately without requiring additional pixel-level occlusion annotations.

To obtain a compact representation for each facial region, global average pooling is applied to each regional feature. The regional descriptor can be written as:


$$\begin{array}{c} z_{i}=GAP(F_{i}),\;i\in \{u,m,d\} \end{array}$$ (7)

where $z_{i}$ denotes the descriptor of the *i*-th facial region, and GAP(·) represents global average pooling. This operation compresses the spatial information of each region and reflects the overall response strength of the corresponding facial area. The three regional descriptors are then organized into a regional feature sequence:


$$\begin{array}{c} Z=[z_{u},z_{m},z_{d}] \end{array}$$ (8)

To generate adaptive regional weights with low computational overhead, an ECA-style one-dimensional convolution is introduced. Compared with a multi-layer perceptron, the 1D convolution can model local dependencies among regional descriptors with fewer parameters. The regional weights are obtained as:


$$\begin{array}{c} \alpha =\sigma \left({Conv1D}_{k}(Z)\right) \end{array}$$ (9)

where ${Conv1D}_{k}(\cdot )$ denotes the one-dimensional convolution with kernel size *k*, σ(·) represents the Sigmoid activation function, and $\alpha =[\alpha_{u},\alpha_{m},\alpha_{d}]$ denotes the adaptive weights of the three facial regions. A larger weight indicates that the corresponding region contains more reliable or discriminative expression information, while a smaller weight suggests that the region may be affected by occlusion, blur, or low-quality features.

Based on the generated regional weights, each facial region is reweighted as follows:


$$\begin{array}{c} F_{i}^{\prime }=\alpha_{i}\cdot F_{i},\;i\in \{u,m,d\} \end{array}$$ (10)

where $F_{i}^{\prime }$ denotes the reweighted feature of the *i*-th region. Through this operation, visible and informative regions are enhanced, while occluded or unreliable regions are suppressed. Finally, the reweighted regional features are concatenated according to their original spatial order to obtain the occlusion-aware enhanced expression feature:


$$\begin{array}{c} F_{orr}=Concat(F_{u}^{\prime },F_{m}^{\prime },F_{d}^{\prime }) \end{array}$$ (11)

where $F_{orr}$ denotes the facial expression feature after regional reweighting. This module does not require additional occlusion masks or complex occlusion restoration operations. Instead, it adaptively adjusts regional contributions at the feature level, enabling the model to focus more on reliable expression cues. Compared with directly using the global backbone feature, the reweighted feature $F_{orr}$ provides a more robust representation for subsequent facial expression classification and emotion intensity prediction.

### Quantitative emotion prediction head

After occlusion-aware regional reweighting, the model obtains the enhanced facial expression feature $F_{orr}$. This feature has been adaptively adjusted according to the reliability of different facial regions, providing a more stable representation for subsequent emotion prediction. Conventional facial expression recognition methods usually output only discrete emotion categories, which makes it difficult to describe the intensity differences within the same expression class. To provide more fine-grained emotional information, a quantitative emotion prediction head is designed to jointly perform facial expression classification and emotion intensity estimation.

First, global average pooling is applied to the reweighted feature to convert the spatial feature map into a compact expression representation:


$$\begin{array}{c} g=GAP(F_{orr}) \end{array}$$ (12)

where *g* denotes the global facial expression feature vector, and GAP(·) represents the global average pooling operation. The prediction feature is formulated according to the input type. For an isolated facial image, the pooled representation is directly used for subsequent prediction, namely *h* = *g*. When temporally continuous video frames are available, the prediction head additionally supports a lightwei*g*ht frame-difference pathway that incorporates short-term variation between adjacent frames. This pathway is an auxiliary architectural extension for sequential video input rather than a heavy temporal modeling component based on recurrent networks or Transformers.

Let $g_{t}$ and $g_{t-1}$ denote the global features of the current frame and the previous frame, respectively. The frame-difference feature is calculated as $g_{t}-g_{t-1}$, which represents the lightweight temporal variation between adjacent frames. To explicitly fuse the current-frame expression representation and the temporal variation cue, a concatenation-based fusion strategy followed by linear projection is adopted:


$$\begin{array}{c} h_{t}=\phi \left(W_{f}[g_{t};g_{t}-g_{t-1}]+b_{f}\right). \end{array}$$ (13)

where $\left[g_{t};g_{t}-g_{t-1}\right]$ denotes feature concatenation along the channel dimension, $W_{f}$ and $b_{f}$ are learnable projection parameters, and ϕ(·) denotes a nonlinear activation function. The fused feature $h_{t}$ contains both the current-frame facial expression representation and the lightweight frame-level temporal variation information. Accordingly, the prediction head follows $h=h_{t}$ when sequential video features are available and *h* = *g* when the input consists of an isolated facial image. Since RAF-DB and AffectNet-7 provide static facial images rather than temporally adjacent frame sequences, the classification and regression experiments reported in this study adopt the static-image formulation *h* = *g*.

In the classification branch, the joint feature *h* is fed into a fully connected layer, followed by a Softmax function to obtain the probability distribution over facial expression categories:


$$\begin{array}{c} p=Softmax(W_{c}h+b_{c}) \end{array}$$ (14)

where *p* denotes the predicted expression probability distribution, and $W_{c}$ and $b_{c}$ are the learnable weight and bias of the classification branch, respectively. This branch is used to predict the basic facial expression category of the input face image or video frame.

In the emotion intensity regression branch, the same joint feature *h* is fed into another fully connected layer to estimate the quantitative emotion intensity score:


$$\begin{array}{c} s=W_{r}h+b_{r} \end{array}$$ (15)

where *s* denotes the predicted emotion intensity score, and $W_{r}$ and $b_{r}$ are the learnable weight and bias of the regression branch, respectively. This branch further describes the activation degree of the facial expression beyond the discrete category prediction, enabling the model to estimate not only which emotion is expressed but also how strongly it is expressed.

To jointly optimize facial expression classification and emotion intensity estimation, a multi-task loss function is adopted. The classification task is optimized using cross-entropy loss, while the emotion intensity regression task is optimized using mean squared error loss in the final experiments. The total loss is defined as:


$$\begin{array}{c} L_{total}=L_{cls}+\lambda L_{reg} \end{array}$$ (16)

where $L_{cls}$ denotes the facial expression classification loss, $L_{reg}$ denotes the emotion intensity regression loss based on mean squared error, and λ is a balancing coefficient that controls the contribution of the regression task. Through this multi-task learning strategy, OQ-FERNet can preserve categorical expression recognition ability while learning emotion intensity variation. Compared with classification-only FER models, the proposed quantitative emotion prediction head provides more fine-grained emotional outputs, making the model more suitable for emotion understanding in real-time human-computer interaction scenarios.

## Experiments

### Datasets and preprocessing

To evaluate the effectiveness of the proposed OQ-FERNet, two widely used in-the-wild facial expression recognition datasets, RAF-DB and AffectNet, are adopted in this study. RAF-DB is used to evaluate facial expression classification performance under unconstrained conditions. It contains facial images with variations in pose, illumination, occlusion, age, gender, and expression ambiguity, which makes it suitable for testing the robustness of facial expression recognition models in real-world scenarios. AffectNet is used to evaluate both facial expression classification and quantitative emotion prediction. In addition to discrete expression labels, AffectNet provides continuous affective annotations, including separate valence and arousal scores. In this study, the arousal annotation is used to construct a scalar supervision target for emotion intensity estimation. The valence–arousal coordinates are not mapped to an intensity vector, and no pre-existing standalone expression-intensity indicator is extracted from the dataset metadata.

For both datasets, seven basic expression categories are selected, including neutral, happiness, sadness, surprise, fear, disgust, and anger. For RAF-DB, the official training and testing split is used. For AffectNet, the seven-class setting is adopted by excluding the contempt category, so that the expression categories are consistent with RAF-DB. For clarity, this seven-class setting of AffectNet is denoted as AffectNet-7 in the following experimental results. The manually annotated subset of AffectNet is used, and the training and validation samples corresponding to the selected seven expression categories are adopted for model training and evaluation.

Before being fed into the network, all facial images are processed using the same preprocessing pipeline. First, face detection and a standard five-landmark-based similarity affine alignment are performed for both RAF-DB and AffectNet-7. Specifically, the centers of the two eyes, the nose tip, and the two mouth corners are detected and mapped to predefined locations in a canonical facial template through translation, in-plane rotation, and isotropic scaling. The aligned facial regions are then cropped and resized to 224×224 pixels. This procedure reduces variations in facial position, scale, and moderate in-plane rotation, thereby improving the spatial consistency of the upper-face, middle-face, and lower-face regions used by the regional reweighting module. Explicit three-dimensional face frontalization is not applied in this study. Therefore, although the landmark-based affine alignment alleviates moderate pose and rotation variations, it cannot completely compensate for extreme out-of-plane head poses or inaccurate landmark localization under severe occlusion. The aligned facial images are normalized using the mean and standard deviation of the training set. During training, data augmentation strategies are applied to improve model generalization, including random horizontal flipping, random cropping, color jittering, and random erasing. During testing, only resizing and normalization are used to ensure stable evaluation.

For the quantitative emotion activation regression task on AffectNet, the arousal annotation is adopted as the regression target. Let $a_{i}\in [-1,1]$ denote the original arousal value of the *i*-th sample. The normalized regression target is obtained through linear transformation as $y_{i}=(a_{i}+1)/2$, where $y_{i}\in [0,1]$. A larger value of $y_{i}$ indicates a higher level of emotional activation, while a smaller value represents a lower activation level. The valence annotation is not used in this task because it reflects the positive-negative direction of emotion rather than activation intensity. Therefore, the regression branch predicts a single normalized emotion activation score instead of a two-dimensional valence-arousal representation.

### Implementation details

All experiments are implemented using PyTorch. The proposed OQ-FERNet and all baseline models are trained under the same preprocessing, data augmentation, and optimization settings to ensure fair comparison. The input image size is fixed to 224×224. The batch size is set to 64, and the total number of training epochs is set to 100. AdamW is used as the optimizer with an initial learning rate of $1\times 10^{-4}$ and a weight decay of $1\times 10^{-4}$. A cosine learning rate decay strategy is adopted to gradually reduce the learning rate during training. For the classification task, cross-entropy loss is used to optimize the facial expression category prediction branch. For the emotion intensity regression task, mean squared error loss is adopted as the default regression loss. The total training objective is defined as a weighted combination of classification loss and regression loss, where the balancing coefficient λ is set to 0.5 unless otherwise specified. Dropout with a probability of 0.3 is used in the prediction head to reduce overfitting. The models are trained on a workstation equipped with an NVIDIA GPU. The software environment includes Python 3.10, PyTorch 2.0, CUDA 11.8, and cuDNN. During inference efficiency evaluation, the batch size is set to 1 to simulate real-time prediction. The inference time and frames per second are measured after a warm-up stage to avoid unstable timing caused by GPU initialization.Since RAF-DB and AffectNet-7 are still-image facial expression recognition datasets, the optional frame-difference branch is not activated in the reported experiments. For all single-image evaluations, the prediction feature is directly set as *h* = *g*, and no temporal difference feature is used. Therefore, the reported classification accuracy, quantitative emotion prediction results, model complexity, FLOPs, inference time, and FPS are all obtained under the static-image setting. The frame-difference feature is only retained as an optional extension for video-based real-time human-computer interaction scenarios.

### Evaluation metrics

The evaluation metrics include both recognition performance and model efficiency. For facial expression classification, Accuracy, Precision, Recall, and F1-score are used. Accuracy measures the overall classification correctness, while Precision, Recall, and F1-score provide a more comprehensive evaluation of class-level prediction quality, especially when the data distribution is imbalanced. Considering the class imbalance in RAF-DB and AffectNet-7, Precision, Recall, and F1-score are calculated using macro averaging across the seven expression categories. Accordingly, the reported values correspond to Macro Precision, Macro Recall, and Macro F1-score. For quantitative emotion prediction, Mean Absolute Error (MAE) and Mean Squared Error (MSE) are used to evaluate the difference between the predicted emotion intensity score and the ground-truth intensity label. Root Mean Squared Error (RMSE) is additionally reported to measure the prediction error in the same numerical scale as the normalized regression target. A lower regression error indicates that the model can estimate emotion intensity more accurately. To account for stochastic variation, all performance-related experiments are independently repeated using five fixed random seeds, namely {0,1,2,3,4}. The dataset partitions, preprocessing procedures, training settings, and optimization parameters remain unchanged across runs. Unless otherwise specified, the results are reported as mean ± standard deviation over the five runs.

To evaluate model complexity and real-time inference capability, the number of parameters, floating-point operations (FLOPs), single-image inference time, and frames per second (FPS) are reported. The number of parameters reflects the storage cost of the model, while FLOPs measure the computational complexity. Inference time and FPS are used to evaluate whether the model satisfies the efficiency requirement of real-time facial expression recognition. Parameters and FLOPs are deterministic structural measurements, whereas inference time and FPS are obtained through repeated runtime measurements after GPU warm-up.

### Baseline models

To evaluate the performance of the proposed OQ-FERNet, several representative baseline models are selected for comparison. The baseline methods include both conventional convolutional networks and recent facial expression recognition models. VGG16 and ResNet18 are adopted as standard CNN-based baselines to provide comparisons with commonly used deep feature extractors. EfficientNet-B0 and ShuffleNetV2 are selected as efficient convolutional models, which are used to evaluate the accuracy-efficiency trade-off of OQ-FERNet. In addition to these general visual recognition backbones, several recent FER methods are included for comparison. CNN-DET [47] is selected as a hybrid deep learning model that combines CNN-based feature extraction with an ensemble classifier. EA-Net [48] is adopted as an attention-enhanced ensemble model, which integrates multiple CNN backbones and attention modules for facial emotion recognition. FERMam [49] is included as a recent lightweight dual-source and multi-scale fusion framework for FER. POSTER-Var [50] is selected as a recent probabilistic FER model based on variational inference, which is used to compare fine-grained expression representation ability. For fair comparison, the original architectural designs and prediction mechanisms of all baseline models were retained. The common preprocessing, training, evaluation, and multi-seed protocols described in the Implementation Details and Evaluation Metrics subsections were consistently applied to all available implementations. No additional model-specific hyperparameter tuning was performed to favor any individual baseline.

## Results

### Comparison results

Table 1 presents the classification performance comparison of different models on the RAF-DB and AffectNet-7 datasets. OQ-FERNet achieves the highest results across all evaluation metrics on both datasets, indicating that the proposed model has effective classification capability and stable recognition performance in facial expression recognition scenarios. On RAF-DB, OQ-FERNet obtains an Accuracy of 93.12%±0.18%, a Precision of 92.84%±0.20%, a Recall of 92.66%±0.19%, and an F1-score of 92.73%±0.17%. Compared with the conventional CNN baselines VGG16 and ResNet18, OQ-FERNet improves Accuracy by 12.70 and 10.21 percentage points, respectively. Compared with EfficientNet-B0 and ShuffleNetV2, the Accuracy improvements are 7.78 and 11.10 percentage points, respectively. These results show that the proposed model can learn more discriminative facial expression representations than basic convolutional and lightweight recognition networks. Compared with CNN-DET on RAF-DB, OQ-FERNet improves Accuracy by 3.42 percentage points and also achieves higher Precision, Recall, and F1-score values. Compared with the stronger models FERMam and POSTER-Var, OQ-FERNet achieves Accuracy improvements of 0.99 and 0.36 percentage points, respectively. Although the margin over POSTER-Var is relatively moderate, the narrow standard deviations confirm that this performance gain reflects a consistent improvement rather than training noise. Furthermore, while achieving superior recognition performance, OQ-FERNet is specifically designed for lightweight and real-time inference. On AffectNet-7, OQ-FERNet obtains an Accuracy of 68.32%±0.23%, a Precision of 67.86%±0.25%, a Recall of 67.41%±0.22%, and an F1-score of 67.58%±0.21%. Compared with VGG16 and ResNet18, OQ-FERNet improves Accuracy by 9.48 and 7.57 percentage points, respectively. Compared with EfficientNet-B0 and ShuffleNetV2, the Accuracy improvements are 5.98 and 8.47 percentage points, respectively. The lower performance on AffectNet-7 than on RAF-DB reflects the greater difficulty of AffectNet-7, which contains more diverse facial appearances, pose variations, illumination changes, expression ambiguity, and label noise. Compared with CNN-DET on AffectNet-7, OQ-FERNet improves Accuracy by 4.90 percentage points. Compared with FERMam and POSTER-Var, OQ-FERNet also achieves higher classification performance, with Accuracy improvements of 1.94 and 0.41 percentage points, respectively. These results indicate that OQ-FERNet can maintain better recognition performance under more challenging in-the-wild conditions. The classification results on both datasets verify the effectiveness of OQ-FERNet and show that occlusion-aware regional reweighting and quantitative prediction design can improve the representation ability for different expression categories.

**Table 1:** Classification performance comparison on RAF-DB and AffectNet-7 (mean ± standard deviation over 5 runs).

Model	RAF-DB				AffectNet-7			
	Accuracy	Precision	Recall	F1-score	Accuracy	Precision	Recall	F1-score
VGG16	80.42±0.35	79.80±0.41	78.95±0.38	79.21±0.36	58.84±0.42	57.96±0.45	57.31±0.40	57.52±0.39
ResNet18	82.91±0.31	82.32±0.35	81.74±0.33	81.96±0.30	60.75±0.38	59.88±0.40	59.40±0.35	59.53±0.36
EfficientNet-B0	85.34±0.28	84.91±0.30	84.28±0.29	84.51±0.27	62.34±0.35	61.75±0.33	61.26±0.31	61.39±0.32
ShuffleNetV2	82.02±0.33	81.58±0.36	80.90±0.34	81.17±0.32	59.85±0.40	58.92±0.42	58.51±0.38	58.63±0.37
CNN-DET	89.70±0.25	89.20±0.27	88.95±0.26	89.04±0.24	63.42±0.31	62.86±0.34	62.35±0.30	62.57±0.29
EA-Net	89.05±0.29	88.60±0.31	88.18±0.28	88.32±0.27	63.80±0.33	63.24±0.35	62.70±0.32	62.91±0.30
FERMam	92.13±0.22	91.80±0.24	91.64±0.23	91.70±0.21	66.38±0.28	65.82±0.30	65.41±0.27	65.55±0.26
POSTER-Var	92.76±0.20	92.43±0.22	92.31±0.21	92.37±0.19	67.91±0.25	67.35±0.27	66.91±0.24	67.05±0.23
OQ-FERNet	93.12±0.18	92.84±0.20	92.66±0.19	92.73±0.17	68.32±0.23	67.86±0.25	67.41±0.22	67.58±0.21

Table 2 presents the quantitative emotion prediction performance of different models on AffectNet-7. Compared with the baseline models, OQ-FERNet achieves the lowest MAE, MSE, and RMSE values, indicating that the proposed model can estimate emotion intensity more accurately. Specifically, OQ-FERNet obtains an MAE of 0.246±0.003, an MSE of 0.101±0.002, and an RMSE of 0.318±0.003, outperforming VGG16, ResNet18, EfficientNet-B0, and ShuffleNetV2 under the same evaluation setting. Among the baseline models, EfficientNet-B0 achieves relatively better quantitative prediction performance, with an MAE of 0.292±0.004, an MSE of 0.139±0.003, and an RMSE of 0.373±0.004. Compared with EfficientNet-B0, OQ-FERNet reduces MAE by 0.046, MSE by 0.038, and RMSE by 0.055. This improvement suggests that the occlusion-aware regional reweighting module can provide more reliable facial representations for emotion intensity estimation by enhancing informative facial regions and suppressing less reliable local features. Compared with ShuffleNetV2, OQ-FERNet also achieves a clear reduction in prediction error, showing that the proposed model can maintain quantitative prediction capability while preserving lightweight inference. These results demonstrate that OQ-FERNet is not limited to discrete facial expression classification, but can also provide fine-grained emotion intensity estimation. This ability is important for real-time human-computer interaction scenarios, where the system needs to understand not only the emotion category but also the strength of the expressed emotion.

**Table 2:** Quantitative emotion prediction performance on AffectNet-7 (mean ± standard deviation over 5 runs).

Model	MAE	MSE	RMSE
VGG16	0.318±0.005	0.159±0.004	0.399±0.006
ResNet18	0.301±0.004	0.147±0.003	0.383±0.005
EfficientNet-B0	0.292±0.004	0.139±0.003	0.373±0.004
ShuffleNetV2	0.309±0.005	0.151±0.004	0.389±0.005
OQ-FERNet	0.246±0.003	0.101±0.002	0.318±0.003

As shown in Table 3 and Fig 2, the proposed OQ-FERNet achieves a favorable balance between recognition accuracy and computational efficiency. Table 3 provides the detailed numerical comparison in terms of parameters, FLOPs, model size, inference time, and FPS, while Fig 2 visualizes the accuracy-efficiency trade-off among different FER models on RAF-DB. In the figure, models located closer to the upper-left region indicate higher recognition accuracy with lower computational cost, and the bubble size represents the inference speed. VGG16 has the highest computational complexity, with 138.36M parameters, 15.47G FLOPs, and a model size of 527.80 MB, while its inference speed is only 78 FPS. Although ShuffleNetV2 achieves the lowest FLOPs and the highest FPS, its recognition accuracy is much lower than that of OQ-FERNet, indicating that excessive lightweight design may weaken facial expression representation capability. FERMam and POSTER-Var obtain relatively strong recognition performance, but they require substantially higher computational costs and show lower inference speed than OQ-FERNet. In contrast, OQ-FERNet achieves an accuracy of 93.12% on RAF-DB with only 4.18M parameters, 0.26G FLOPs, and 16.10 MB model size. Its inference time is 2.81 ms, corresponding to 356 FPS, which satisfies the requirement of real-time facial expression recognition. These results indicate that OQ-FERNet does not improve recognition performance by increasing computational complexity. Instead, by combining lightweight feature extraction with occlusion-aware regional reweighting and quantitative prediction design, the proposed model achieves a better trade-off between accuracy and efficiency. Therefore, OQ-FERNet is more suitable for real-time human-computer interaction scenarios where both recognition performance and inference speed are required.

**Table 3:** Model complexity and inference efficiency.

Model	Params / M	FLOPs / G	Model Size / MB	Inference Time / ms	FPS
VGG16	138.36	15.47	527.80	12.82	78
ResNet18	11.69	1.82	44.60	4.08	245
EfficientNet-B0	5.29	0.39	20.20	3.23	310
ShuffleNetV2	2.28	0.15	8.70	2.33	430
CNN-DET	18.62	3.94	71.05	7.84	128
EA-Net	30.40	6.25	116.00	10.53	95
FERMam	26.99	5.97	103.00	7.20	139
POSTER-Var	43.80	8.45	167.00	10.98	91
OQ-FERNet	4.18	0.26	16.10	2.81	356

**Fig 2:**
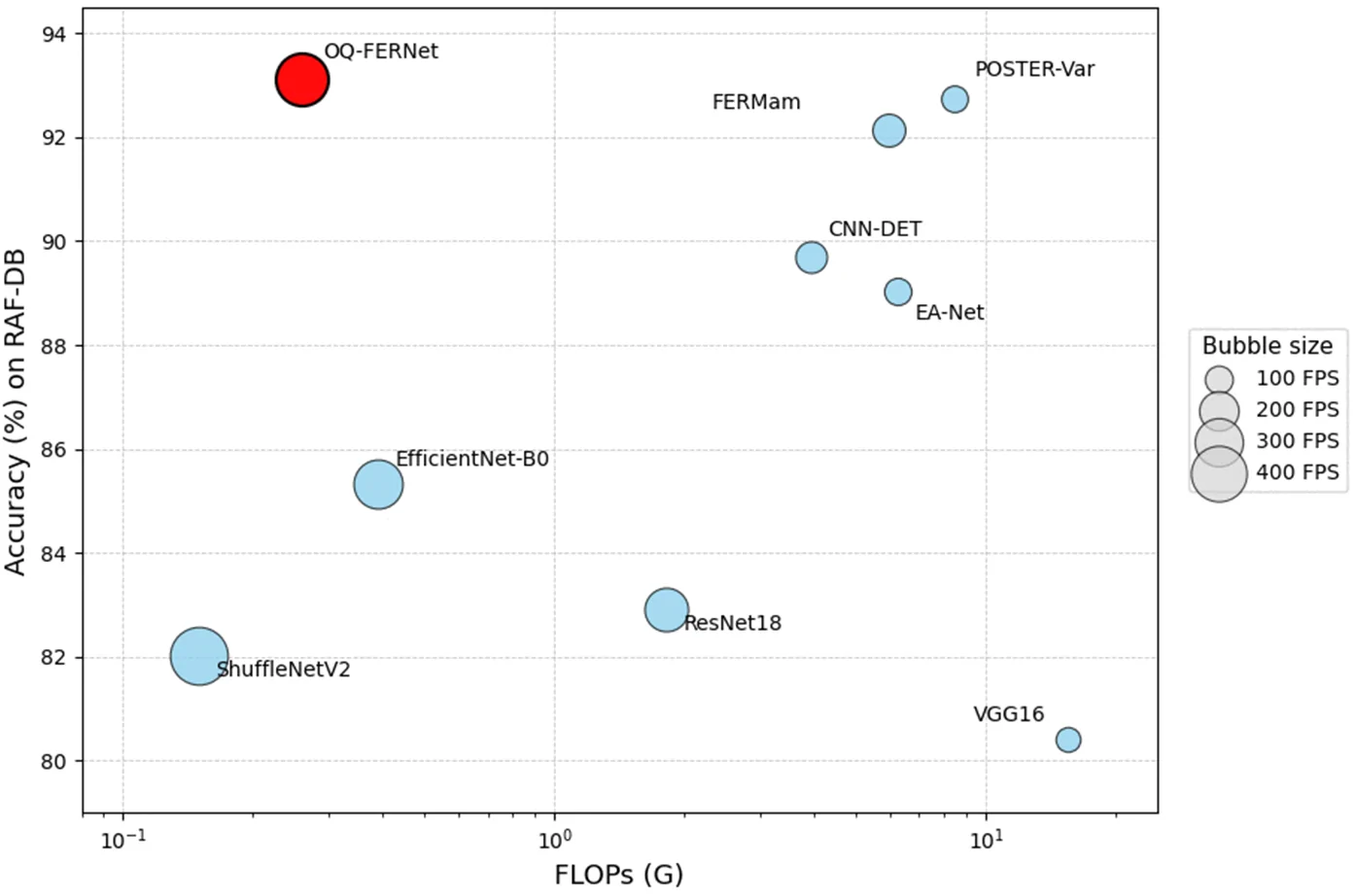
Accuracy-efficiency comparison of different FER models on RAF-DB.

For robustness evaluation, a controlled synthetic occlusion protocol is designed on aligned RAF-DB images. Three occlusion conditions are considered, including eye occlusion, mouth occlusion, and random occlusion. Eye occlusion and mouth occlusion are generated by masking the corresponding facial regions after alignment, while random occlusion is generated by applying masks at randomly selected locations within the facial area. The same occlusion generation strategy is applied to all models to ensure a fair and reproducible comparison.

Table 4 presents the robustness comparison of different models under synthetic occlusion on RAF-DB. The no-occlusion results are consistent with the RAF-DB classification results reported in Table 1. Under the no-occlusion setting, OQ-FERNet achieves an accuracy of 93.12%±0.18%, outperforming all compared models. When different types of occlusion are introduced, the proposed model still maintains the highest accuracy, reaching 89.62%±0.23% under eye occlusion, 88.73%±0.21% under mouth occlusion, and 88.96%±0.22% under random occlusion. Compared with VGG16, ResNet18, EfficientNet-B0, and ShuffleNetV2, OQ-FERNet shows a much smaller performance degradation, indicating that the proposed model is more stable when local facial information is partially missing. Compared with CNN-DET and EA-Net, OQ-FERNet also achieves higher accuracy under all occlusion settings. In particular, the average drop of OQ-FERNet is only 4.02%±0.16%, which is lower than CNN-DET and EA-Net by 4.63 and 3.73 percentage points, respectively. Compared with FERMam and POSTER-Var, OQ-FERNet also obtains a smaller average drop, showing better robustness against synthetic occlusion. These results suggest that the occlusion-aware regional reweighting module can reduce the negative influence of unreliable facial regions and improve the stability of expression recognition under degraded visual conditions. Fig 3 further visualizes the average regional weights generated by the ORR module under different occlusion conditions. Under the no-occlusion setting, the weights of the upper-face, middle-face, and lower-face regions are relatively balanced, indicating that the model jointly uses information from different facial regions. Under eye occlusion, the upper-face weight decreases to 0.18, while the lower-face weight increases to 0.47. This indicates that the model suppresses the occluded upper-face region and relies more on visible regions. Under mouth occlusion, the lower-face weight decreases to 0.19, while the upper-face weight increases to 0.46, showing that the model shifts attention away from the occluded mouth region. Under random occlusion, the three regional weights remain relatively balanced, reflecting the adaptive response of the model to uncertain local information loss. The results provide consistent evidence for the effectiveness of the ORR module. The accuracy comparison shows that OQ-FERNet achieves the smallest performance degradation under occlusion, while the regional weight visualization explains how the model adjusts the contributions of different facial regions. These results demonstrate that the proposed regional reweighting mechanism can adaptively suppress unreliable occluded regions and enhance informative visible regions, thereby improving the occlusion robustness of facial expression recognition. Although the synthetic occlusion protocol provides a controlled evaluation of regional information loss, it relies on the accuracy of facial alignment. Landmark localization errors, extreme head poses, or large facial rotations may reduce the correspondence between predefined regions and actual facial components, which may affect the robustness evaluation. In addition, RAF-DB and AffectNet-7 contain naturally occurring occlusions as part of their unconstrained variations; however, their standard annotations do not provide independent labels for specific natural occlusion categories, such as masks, glasses, hair, or hand occlusions. Therefore, the current evaluation focuses on controlled synthetic occlusion to ensure reproducibility, while future work will investigate adaptive facial normalization, learnable regional modeling, and independently annotated natural-occlusion benchmarks to further improve generalization under realistic conditions.

**Table 4:** Robustness comparison under synthetic occlusion on RAF-DB.

Model	No Occlusion	Eye Occlusion	Mouth Occlusion	Random Occlusion	Average Drop
VGG16	80.42±0.35	71.48±0.42	68.95±0.39	70.62±0.40	10.07±0.32
ResNet18	82.91±0.31	74.85±0.38	71.96±0.35	73.50±0.36	9.47±0.29
EfficientNet-B0	85.34±0.28	77.83±0.35	75.16±0.32	76.34±0.33	8.90±0.26
ShuffleNetV2	82.02±0.33	73.40±0.40	70.25±0.37	72.06±0.38	10.12±0.30
CNN-DET	89.70±0.25	82.76±0.31	80.48±0.28	81.92±0.29	7.98±0.24
EA-Net	89.05±0.29	82.40±0.33	80.16±0.30	81.35±0.31	7.75±0.25
FERMam	92.13±0.22	87.70±0.28	86.21±0.25	86.95±0.26	5.18±0.20
POSTER-Var	92.76±0.20	88.10±0.25	86.58±0.23	87.33±0.24	5.42±0.18
OQ-FERNet	93.12±0.18	89.62±0.23	88.73±0.21	88.96±0.22	4.02±0.16

**Fig 3:**
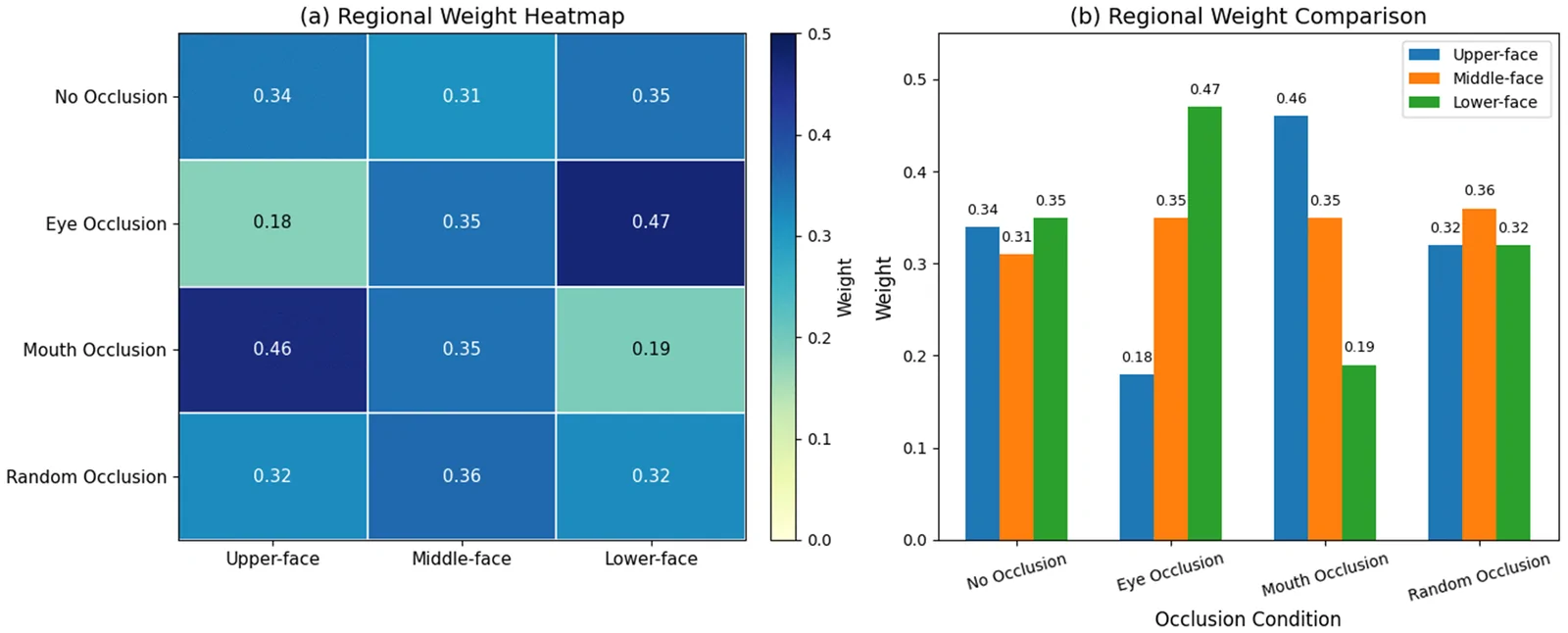
Regional weight visualization of the ORR module on RAF-DB under different synthetic occlusion conditions.

Fig 4 shows the normalized confusion matrices of OQ-FERNet on RAF-DB and AffectNet-7. The rows represent the ground-truth expression labels, and the columns represent the predicted labels. Since the matrices are normalized by rows, each diagonal entry indicates the proportion of correctly classified samples within the corresponding ground-truth category. On RAF-DB, most samples are concentrated along the diagonal, indicating that OQ-FERNet can effectively distinguish the seven basic facial expression categories. The model achieves high diagonal proportions for Happiness, Surprise, Anger, and Neutral, with values of 97.2%, 93.6%, 93.4%, and 93.1%, respectively. This suggests that OQ-FERNet can capture discriminative facial cues for expressions with relatively clear visual patterns. For RAF-DB, the relatively lower diagonal entries for Fear and Disgust indicate that these two categories are more difficult to distinguish than Happiness and Surprise. Disgust is partly confused with Anger, while Fear is partly confused with Surprise, reflecting the visual similarity between these expression pairs. This phenomenon is consistent with the fact that negative expressions often share subtle local facial movements, such as changes around the eyes, nose, and mouth corners. On AffectNet-7, the diagonal entries are generally lower than those on RAF-DB, which reflects the greater difficulty of AffectNet-7 under in-the-wild conditions. Although Happiness still shows a relatively high diagonal proportion, categories such as Fear, Disgust, Sadness, and Anger exhibit more visible off-diagonal confusion. In particular, Fear is more likely to be confused with Surprise, and Disgust is more likely to be confused with Anger. This indicates that AffectNet-7 contains more challenging samples with larger variations in pose, illumination, expression intensity, and annotation ambiguity. The confusion matrices further support the quantitative results reported in Table 1. RAF-DB shows stronger class-wise separability, while AffectNet-7 presents more complex inter-class confusion. Nevertheless, OQ-FERNet maintains clear diagonal dominance on both datasets, demonstrating that the proposed occlusion-aware regional reweighting and quantitative prediction design can enhance discriminative facial expression representation under different data conditions.

**Fig 4:**
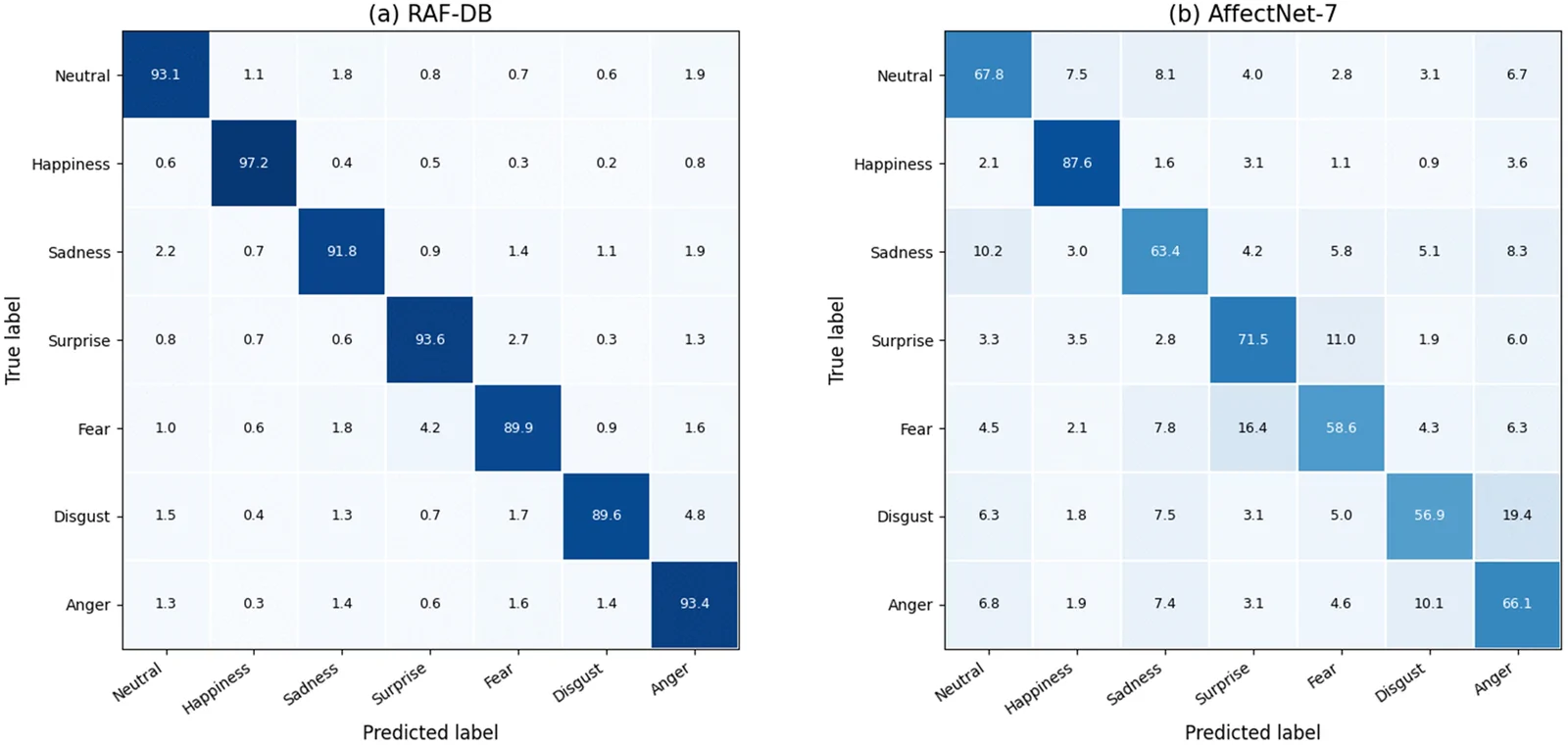
Normalized confusion matrices of OQ-FERNet on RAF-DB and AffectNet.

### Ablation study

Table 5 presents the ablation results of OQ-FERNet on RAF-DB and AffectNet-7. When both regional reweighting and quantitative emotion prediction are removed, the model obtains the lowest classification performance, with 89.42%±0.28% accuracy and 88.71%±0.26% F1-score on RAF-DB, as well as 63.86%±0.35% accuracy and 63.12%±0.33% F1-score on AffectNet-7. This result shows that directly using the basic facial expression feature without regional enhancement and quantitative supervision is insufficient for robust expression representation. After introducing the regional reweighting module, the accuracy increases to 91.05%±0.24% on RAF-DB and 66.74%±0.30% on AffectNet-7. The MAE and MSE on AffectNet-7 are also reduced to 0.263±0.004 and 0.109±0.003, respectively. These results indicate that region-level feature adjustment can enhance informative facial regions and suppress less reliable local features, thereby improving both expression classification and emotion intensity estimation. When the quantitative emotion prediction head is removed, the model achieves 91.62%±0.22% accuracy and 90.91%±0.20% F1-score on RAF-DB, as well as 66.38%±0.28% accuracy and 65.79%±0.26% F1-score on AffectNet-7. Since this variant does not output emotion intensity, MAE and MSE are not applicable. Compared with this variant, the complete OQ-FERNet improves the accuracy from 91.62% to 93.12% on RAF-DB and from 66.38% to 68.32% on AffectNet-7. This demonstrates that quantitative emotion prediction provides useful auxiliary supervision for learning more fine-grained affective representations. The complete OQ-FERNet achieves the best classification results on both datasets and obtains the lowest MAE and MSE values on AffectNet-7, reaching 0.246±0.003 and 0.101±0.002, respectively. These results verify the effectiveness of the occlusion-aware regional reweighting module and the quantitative emotion prediction head. The ablation study shows that the two components are complementary and jointly contribute to improving the classification accuracy, robustness, and fine-grained emotion prediction capability of OQ-FERNet.

**Table 5:** Ablation study of OQ-FERNet.

Model Variant	RAF-DB		AffectNet-7			
	Accuracy / %	F1-score / %	Accuracy / %	F1-score / %	MAE	MSE
w/o Regional Reweighting and Quantitative Emotion Prediction	89.42±0.28	88.71±0.26	63.86±0.35	63.12±0.33	–	–
w/o Regional Reweighting	91.05±0.24	90.34±0.22	66.74±0.30	66.12±0.28	0.263±0.004	0.109±0.003
w/o Quantitative Emotion Prediction	91.62±0.22	90.91±0.20	66.38±0.28	65.79±0.26	–	–
OQ-FERNet	93.12±0.18	92.73±0.17	68.32±0.23	67.58±0.21	0.246±0.003	0.101±0.002

### Sensitivity analysis

Table 6 presents the sensitivity analysis of the multi-task loss weight λ. The parameter λ controls the relative contribution of the emotion intensity regression loss in the total training objective. When λ is set to 0.1, the model obtains 92.31%±0.24% Accuracy and 91.86%±0.22% F1-score on RAF-DB, as well as 67.28%±0.31% Accuracy and 66.63%±0.29% F1-score on AffectNet-7. As λ increases from 0.1 to 0.5, the classification performance improves steadily on both datasets. Specifically, the Accuracy increases from 92.31% to 93.12% on RAF-DB and from 67.28% to 68.32% on AffectNet-7. This indicates that an appropriate regression supervision signal can help the model learn more fine-grained facial expression representations. When λ is further increased to 0.7 and 1.0, the regression errors on AffectNet-7 continue to decrease, with MAE decreasing from 0.246 to 0.241 and MSE decreasing from 0.101 to 0.098. However, the classification performance begins to decline. For example, when λ is set to 1.0, the Accuracy decreases to 92.27%±0.23% on RAF-DB and 67.46%±0.28% on AffectNet-7. This suggests that an excessively large regression weight may make the model overly biased toward emotion intensity estimation, thereby weakening its discriminative ability for discrete expression classification. Therefore, λ=0.5 is selected as the default setting in this study. Under this setting, OQ-FERNet achieves the best classification performance on both RAF-DB and AffectNet-7, while still maintaining low MAE and MSE values for quantitative emotion prediction. The results show that a balanced multi-task optimization strategy is beneficial for improving both facial expression classification and emotion intensity estimation.

**Table 6:** Sensitivity analysis of multi-task loss weight.

Loss Weight λ	RAF-DB		AffectNet-7			
	Accuracy / %	F1-score / %	Accuracy / %	F1-score / %	MAE	MSE
0.1	92.31±0.24	91.86±0.22	67.28±0.31	66.63±0.29	0.260±0.004	0.110±0.003
0.3	92.76±0.21	92.26±0.19	67.91±0.27	67.16±0.25	0.252±0.003	0.105±0.002
0.5	93.12±0.18	92.73±0.17	68.32±0.23	67.58±0.21	0.246±0.003	0.101±0.002
0.7	92.84±0.20	92.39±0.18	68.05±0.25	67.31±0.23	0.243±0.003	0.099±0.002
1.0	92.27±0.23	91.88±0.21	67.46±0.28	66.82±0.26	0.241±0.002	0.098±0.002

## Discussion

The proposed OQ-FERNet is designed to address three practical requirements in facial expression recognition: lightweight inference, robustness to partial occlusion, and fine-grained emotion representation. Compared with conventional classification-only FER models, OQ-FERNet introduces an occlusion-aware regional reweighting mechanism and a quantitative emotion prediction head. These two designs allow the model to focus on more reliable facial regions and provide emotion intensity estimation in addition to discrete expression categories. The regional reweighting mechanism provides an interpretable way to improve robustness under occlusion. Instead of treating the whole face as a single global representation, OQ-FERNet divides facial features into upper-face, middle-face, and lower-face regions. This structure is consistent with the fact that different expressions are often reflected by different local facial areas, such as the eyes, eyebrows, cheeks, and mouth. When partial occlusion occurs, the model can reduce the contribution of unreliable regions and rely more on visible facial cues. Therefore, the proposed module improves not only recognition performance but also the interpretability of the decision process. Recent FER studies have explored different strategies to improve recognition performance under unconstrained conditions, including attention-based feature enhancement, multi-scale representation learning, and probabilistic expression modeling [49,50]. However, many existing approaches mainly focus on improving global feature discrimination or expression representation capability, while the robustness of local facial regions under partial occlusion remains insufficiently considered. In contrast, OQ-FERNet explicitly introduces regional reliability modeling and adaptive feature reweighting, which provides a complementary perspective for improving occlusion robustness. The experimental results on RAF-DB and AffectNet-7 demonstrate that the proposed strategy achieves competitive recognition performance compared with recent FER methods while maintaining a lightweight computational structure. The quantitative prediction head extends the output of OQ-FERNet from categorical expression recognition to fine-grained emotion intensity estimation. This is important for real-time human-computer interaction, because an interactive system often needs to understand not only which emotion is expressed but also how strongly it is expressed. By jointly optimizing classification and regression objectives, the model can learn more informative affective representations. This design makes OQ-FERNet more suitable for emotion-aware applications that require continuous or intensity-based feedback.

Although OQ-FERNet achieves a favorable balance among accuracy, robustness, and computational efficiency, several limitations should be acknowledged. One limitation concerns the use of three fixed horizontal facial regions. Before feature extraction, a standard five-landmark-based similarity affine transformation is applied to the RAF-DB and AffectNet-7 images. This alignment procedure reduces variations in facial position, scale, and moderate in-plane rotation, thereby improving the spatial correspondence between the predefined feature regions and the underlying facial components. However, explicit three-dimensional face frontalization is not performed. Consequently, extreme out-of-plane poses, pronounced head tilt, severe facial rotation, or inaccurate landmark localization under heavy occlusion may still disrupt the correspondence between the horizontal feature slices and the actual upper-, middle-, and lower-face regions. Furthermore, although RAF-DB and AffectNet-7 contain naturally occurring occlusions as part of their unconstrained visual variability, the standard dataset partitions used in this study do not provide separate fine-grained annotations for specific real-world occlusion types, such as medical masks, sunglasses, hair, or hand occlusion. Therefore, the corresponding samples cannot be reliably and reproducibly disaggregated into dedicated natural-occlusion subsets under the current evaluation protocol without introducing an additional manual annotation procedure. The dedicated occlusion robustness analysis in this study consequently adopts controlled synthetic eye, mouth, and random occlusions to isolate regional information loss and ensure consistent comparisons across different models. Nevertheless, synthetic geometric occlusions cannot fully reproduce the irregular shapes, textures, reflections, landmark disturbances, and pose–occlusion interactions encountered in practical environments. A further limitation is that quantitative emotion prediction is evaluated using the continuous affective annotations of AffectNet only. Additional evaluation on datasets containing compatible continuous emotion annotations would be beneficial for establishing the generalization capability of the regression branch.

Future work will investigate more adaptive facial normalization and regional modeling strategies. Three-dimensional face frontalization, landmark-guided regional extraction, and learnable region generation may improve the model’s ability to handle extreme pose variations, facial rotation, and non-rigid deformation. The robustness evaluation will also be extended to independently annotated and publicly reproducible natural-occlusion benchmarks containing real masks, sunglasses, hair, hands, and mixed occlusion conditions, thereby providing a more realistic assessment under practical environments. The existing frame-difference pathway already provides a lightweight first-order temporal cue by modeling feature variations between two adjacent frames. Building on this auxiliary mechanism, future work will investigate compact GRU-based aggregation and lightweight temporal Transformers to capture longer-range dependencies across multiple frames. The reported 356 FPS represents the current static-image inference efficiency and will serve as a computational reference rather than a guaranteed throughput for the extended sequence models. Future temporal architectures will therefore be jointly assessed in terms of recognition performance, parameter count, FLOPs, latency, and FPS to preserve practical real-time processing capability on edge hardware.

## Conclusion

This paper presents OQ-FERNet, an occlusion-aware quantitative facial expression recognition network for real-time human-computer interaction scenarios. The model combines lightweight feature extraction, regional feature reweighting, and quantitative emotion prediction in a unified framework. By adaptively adjusting the contributions of different facial regions, OQ-FERNet can reduce the influence of occluded or unreliable areas and enhance discriminative expression-related cues. By introducing the quantitative prediction head, the model can further estimate emotion intensity beyond discrete expression classification. Experiments on RAF-DB and AffectNet under the seven-class setting demonstrate that OQ-FERNet can achieve competitive recognition performance while maintaining low computational complexity. The robustness and ablation results further verify the effectiveness of the regional reweighting module and the quantitative prediction design. These findings indicate that OQ-FERNet is suitable for lightweight, occlusion-robust, and fine-grained facial expression recognition.

In future research, OQ-FERNet can be extended through more adaptive facial region modeling, systematic evaluation on real-world occlusion data, and longer-range temporal aggregation built upon the existing lightweight frame-difference pathway. These extensions may further improve the robustness, interpretability, and practical applicability of the proposed framework in real-time human-computer interaction systems.
